# Measurement of Sarcopenia in Head and Neck Cancer Patients and Its Association With Frailty

**DOI:** 10.3389/fonc.2022.884988

**Published:** 2022-05-12

**Authors:** Remco de Bree, Christiaan D. A. Meerkerk, Gyorgy B. Halmos, Antti A. Mäkitie, Akihiro Homma, Juan P. Rodrigo, Fernando López, Robert P. Takes, Jan B. Vermorken, Alfio Ferlito

**Affiliations:** ^1^ Department of Head and Neck Surgical Oncology, University Medical Center Utrecht, University of Utrecht, Utrecht, Netherlands; ^2^ Department of Otorhinolaryngology – Head and Neck Surgery, University Medical Center Groningen, University of Groningen, Groningen, Netherlands; ^3^ Department of Otorhinolaryngology – Head and Neck Surgery, University of Helsinki and Helsinki University Hospital, Helsinki, Finland; ^4^ Department of Otolaryngology - Head and Neck Surgery, Faculty of Medicine and Graduate School of Medicine, Hokkaido University, Sapporo, Japan; ^5^ Department of Otorhinolaryngology - Head and Neck Surgery, Hospital Universitario Central de Asturias, Oviedo, Spain; ^6^ Department of Otolaryngology - Head and Neck Surgery, Radboud University Medical Center, Nijmegen, Netherlands; ^7^ Department of Medical Oncology, Antwerp University Hospital, Edegem, Belgium and Faculty of Medicine and Health Sciences, University of Antwerp, Antwerp, Belgium; ^8^ Coordinator of the International Head and Neck Scientific Group, Padua, Italy

**Keywords:** head and neck cancer, elderly, sarcopenia, skeletal muscle mass, frailty, toxicity, complications, survival

## Abstract

In head and neck cancer (HNC) there is a need for more personalized treatment based on risk assessment for treatment related adverse events (i.e. toxicities and complications), expected survival and quality of life. Sarcopenia, defined as a condition characterized by loss of skeletal muscle mass and function, can predict adverse outcomes in HNC patients. A review of the literature on the measurement of sarcopenia in head and neck cancer patients and its association with frailty was performed. Skeletal muscle mass (SMM) measurement only is often used to determine if sarcopenia is present or not. SMM is most often assessed by measuring skeletal muscle cross-sectional area on CT or MRI at the level of the third lumbar vertebra. As abdominal scans are not always available in HNC patients, measurement of SMM at the third cervical vertebra has been developed and is frequently used. Frailty is often defined as an age-related cumulative decline across multiple physiologic systems, with impaired homeostatic reserve and a reduced capacity of the organism to withstand stress, leading to increased risk of adverse health outcomes. There is no international standard measure of frailty and there are multiple measures of frailty. Both sarcopenia and frailty can predict adverse outcomes and can be used to identify vulnerable patients, select treatment options, adjust treatments, improve patient counselling, improve preoperative nutritional status and anticipate early on complications, length of hospital stay and discharge. Depending on the definitions used for sarcopenia and frailty, there is more or less overlap between both conditions. However, it has yet to be determined if sarcopenia and frailty can be used interchangeably or that they have additional value and should be used in combination to optimize individualized treatment in HNC patients.

## Introduction

Head and neck cancer (HNC) encompasses a heterogeneous group of malignancies that arise in the mucosal linings of the sinonasal cavities, oral cavity, pharynx and larynx. Most HNC patients present with locoregionally advanced disease. Combining various types of treatments - such as surgery, radiation, and chemotherapy - often are required for cure to be achieved. Many of these treatments and procedures can cause toxicity and complications, potentially limiting oncological outcomes. Despite important developments in diagnostics and immune checkpoint inhibitors are the therapeutic highlight of the past decade, survival rates overall in patients with HNC have not dramatically changed (1). New strategies are needed to shift the treatment paradigm from treatment for all patients according to clinical and histological features to personalized treatment guided by biomarkers that identify individual differences between patients (2).

Patients are increasingly being diagnosed with HNC at an older age. In older patients the treatment is complicated by the heterogeneous aging process and associated with a wide diversity in treatment tolerability. There is a need for personalized treatment based on risk assessment for treatment related (dose limiting) toxicities and complications, expected survival and quality of life (3–5). Several patient and tumor characteristics have been implicated in poor tolerance to treatment, such as advanced age, low socio-economic status, advanced clinical stage, liver and kidney disease and poor functional status. However, these risk factors are difficult to modify prior to treatment. The characteristics that can be modified prior to treatment are few. Assessment of frailty and, more recently, sarcopenia have potential predictive and prognostic value in HNC patients and these features could be used to tailor treatment (2, 6). Early identification of those patients who may tolerate treatment poorly may allow for treatment modification and guide future research in these higher risk populations.

The aim of the present review is to describe the different measurement methods to diagnose sarcopenia, its predictive value and its association with frailty.

## Sarcopenia

Sarcopenia lends its name from the Greek words ‘‘sarx’’ meaning flesh and ‘‘penia’’ meaning lack (7). Sarcopenia was first described as the phenomenon of skeletal muscle mass (SMM) loss related to increasing age, while fat remains equal or increases, combined with loss of muscle function (8). Sarcopenia can occur across all body mass index (BMI) categories (9). Although sarcopenia is primary due to ageing, it can also occur secondary due to an underlying disease. The proposed definition of sarcopenia of the European Working Group on Sarcopenia in Older People (EWGSOP) requires a decrease in SMM combined with a decrease in muscle function (10, 11). The Sarcopenia Definition and Outcomes Consortium (SDOC) and the Special Interest Group (SIG) on cachexia-anorexia in chronic wasting diseases (of the European Society for Clinical Nutrition and Metabolism; ESPEN) support the use of both SMM and muscle function for defining sarcopenia (12, 13). However, muscle function is not frequently measured, whereas SMM can often be retrospectively determined. Therefore, despite the importance of decreased muscle function to diagnose sarcopenia, the terms “sarcopenia” and “low SMM” are often used interchangeably in literature.

It is estimated that the prevalence of primary sarcopenia in the general population is 5-13% for people aged 60-70 years, and up to 50% for those aged 80 years or above (14). Sarcopenia is a risk factor for various adverse outcomes such as physical disability, decreased quality of life, and ultimately early death (10). Sarcopenia can also be secondary to chronic systemic inflammation, malnutrition and immobilization, regardless of age (9). While the presence of sarcopenia has been associated with adverse outcomes in numerous chronic diseases, the impact of sarcopenia in oncology has been increasingly appreciated and considered of importance. Cancer patients are generally exposed to several cancer-specific and non-cancer-specific factors that cause a decrease in muscle mass and function. These factors include age and comorbidities, malnutrition, physical inactivity, tumor-derived factors, cancer therapy and supportive medication (15). Moreover, chronic inflammation triggered by the tumor is also an added risk factor for sarcopenia (10–13).

The increase in research related to sarcopenia in cancer has been augmented by the widespread availability of radiology images obtained as part of routine oncology care.

HNC patients are particularly at risk for low SMM due to the location of the tumor which frequently leads to dysphagia and consequently to malnutrition and a catabolic state. At diagnosis, up to 50% of patients with HNC present with signs of malnutrition (16). HNC patients with dysphagia have a lower body mass index (BMI), lower SMM and more often sarcopenia as compared to HNC patients without dysphagia (17). Sarcopenia affects also swallowing-related muscles leading to decreased swallowing function: sarcopenic dysphagia (18). This vicious circle may accelerate the severity of sarcopenia. Evidence is mounting that an abnormal body composition, in particular a low SMM, is an adverse predictive and prognostic factor in HNC patients (2, 19).

### Skeletal Muscle Mass Measurement

Techniques to measure body composition and SMM include ‘dual-energy X-ray’-absorptiometry (DEXA) scan, bioelectrical impedance analysis (BIA), magnetic resonance imaging (MRI) and computed tomography (CT) (9, 20–22). To date, CT is probably the easiest and most promising modality, although limited by the time needed for muscle segmentation (20). However, the evolution of automated CT segmentation to assess body composition will accelerate body composition research and, eventually, facilitate integration of body composition measures into clinical care (23, 24).

**Figure 1:**
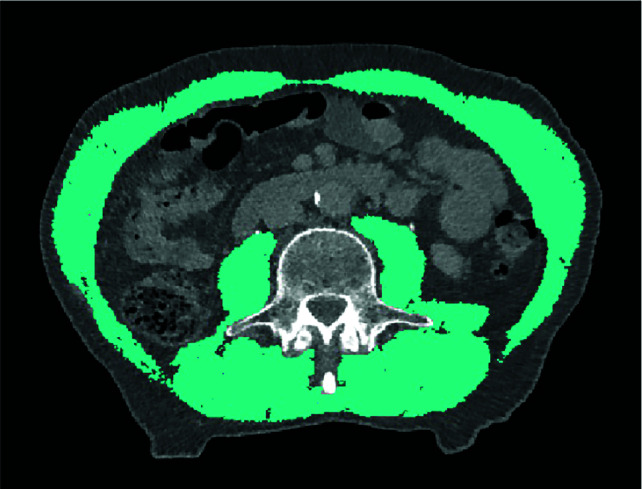
Delineation of skeletal muscle tissue on transversal CT imaging at the level of L3. A Hounsfield Unit window of -29 to +150 was used to accentuate skeletal muscle tissue.

A high correlation between the cross-sectional skeletal muscle area (CSMA) on a single MRI slice at the level of the third lumbar vertebra (L3) and whole-body total skeletal muscle volume as measured on whole-body MRI was found (25). In contrast to SMM measurement on MRI, in which SMM measurement is fully manually performed, measurement on CT imaging can be performed using semi-automatic software programs with predefined Hounsfield unit range (-29 and +150) which is muscle specific (26). Therefore, CSMA measurement on CT at level L3 became the most frequently used measurement method of SMM. The area of the psoas, erector spinae, quadratus lumborum, transversus abdominis, external and internal obliques and rectus abdominis muscles are segmented on a single axial-slice to measure CSMA on this level (**Figure 1**). Because a linear relationship between a person’s height and the skeletal muscle area at the level of L3 was found, CSMA at level of L3 is adjusted for squared height, to calculate the skeletal muscle index (SMI; cm^2^/m^2^), as an estimation of a person’s total SMM in proportion to stature (27).

#### Assessment of Cross-Sectional Skeletal Muscle Area at the Level of the Third Cervical Vertebra

**Figure 2:**
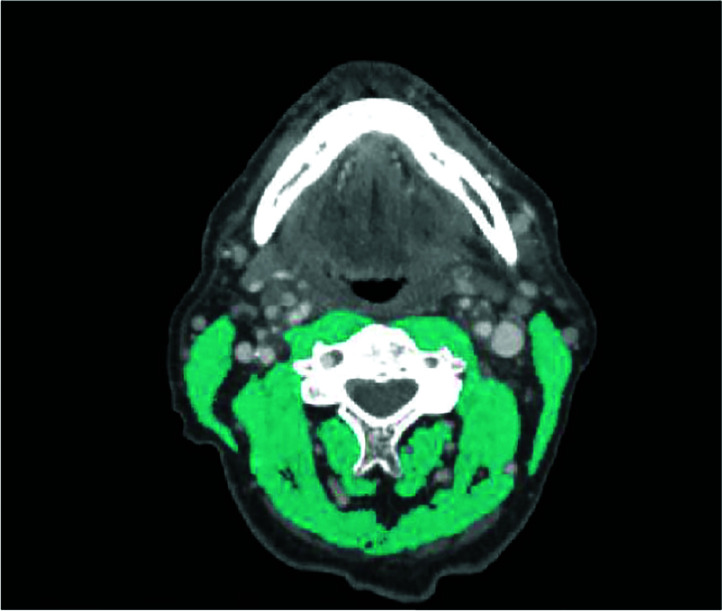
Delineation of skeletal muscle tissue on transversal CT imaging at the level of C3. A Hounsfield Unit window of -29 to +150 was used to accentuate skeletal muscle tissue.

Abdominal CT imaging is not routinely performed in HNC patients and is often only available in patients with locally advanced disease in the context of staging. In 2016, Swartz et al. (28) published an assessment method for SMM using a single CT slice at the level of the third cervical vertebra (C3), which is featured on regular head and neck CT imaging. In this method SMM is assessed at the level of C3 in which both sternocleidomastoid muscles and the paravertebral muscles are segmented (**Figure 2**). In a next step, a good correlation between CSMA at the level of C3 and L3 was found (r = 0.785). A multivariate formula to estimate the CSMA at the level of L3 from the CSMA at the level of C3 was formulated and included gender, age, and weight; the correlation between the estimated CSMA at the level of L3 and the actual CSMA at the level of L3 was excellent (r = 0.891) (28). This SMM assessment at the level of C3 was recently validated in 200 patients with HNC and showed again a good correlation between CSMA at the level of C3 and L3 (r = 0.75). With the use of the multivariate formula to estimate CSMA at L3 the correlation further improved (r = 0.82). Finally, there proved to be a very adequate agreement between the estimated and the actual CSMA at L3 (interclass coefficient (ICC) 0.78). Using a previously defined cut-off value of 43.2 cm^2^/m^2^ for lumbar SMI (29), a moderate agreement in the identification of patients with low SMI based on the estimated lumbar SMI and actual lumbar SMI was found: κ 0.57, 95%CI: 0.45-0.69 (30). Interobserver agreement for CSMA measurement at the level of C3 was investigated in 54 locally advanced HNSCC patients and found to be excellent (ICC 0.763-0.969) for 6 observers (31). In a study of Zwart et al. the excellent interobserver agreement for cross-sectional measurements was confirmed (ICC 0.931-0.982) and an excellent intra-observer agreement was found (ICC 0.957-0.998) (32). The strong correlation of a prediction model based on C3 SMM measurement with the actual L3 SMM was confirmed in a study of 305 advanced stage HNSCC patients (33). Lu et al. developed another prediction formula model in 200 Chinese oral squamous cell carcinoma patients. Adding gender and weight (not age) improved the correlation between estimated and measured L3 CSMA from 0.810 to 0.975 (34). Looking for alternative cervical levels, in 159 HNC patients who underwent PET/CT for tumor staging paravertebral and sternocleidomastoid muscle areas at C2, C3, C4 and L3 were measured. Although SMI at C2, C3 and C4 all showed very strong and significant correlation with SMI at L3 (p < 0.001), the best discriminative for low SMM was SMI at C3 (35).

Yoshimura et al. compared the sternocleidomastoid muscle mass index and psoas muscle mass index by assessment of cross-sectional areas at the levels of C3 and L3, respectively, and found a moderate correlation (r = 0.546; p < 0.0001) (36). Recently Yoon et al. (37) examined the predictive value of CSMA at C3 (without sternocleidomastoid muscle) for CSMA at L3 in 165 HNC patients and 42 healthy adults and found a strong correlation in both healthy adults (r = 0.864) and non-sarcopenic (normal SMI) patients (r = 0.876), while a fair association was found in sarcopenic (low SMI) patients (r = 0.381). A prediction model, including age, sex, weight, showed a very strong correlation between actual SMM at the level of L3 and predicted SMM at the level of L3 in both non-sarcopenic patients and healthy adults (r > 0.9), whereas the relationship was moderate in sarcopenic patients (r = 0.763). Sarcopenia was defined as SMI at L3 < 49 cm^2^/m^2^ for men and < 31 cm^2^/m^2^ for women based on an epidemiological study with a Korean population. They conclude that SMM at the level of C3 may not be a strong predictor for SMM at the level of L3 in sarcopenic HNC patients (37). However, It is likely that there are inter-ethnic differences in skeletal muscle mass assessment. In a cohort of 200 Dutch patients CSMA at C3 and L3 correlated well in patients with normal as well as low SMM. In the 77 patients with low SMM (lumbar SMI ≤ 43.2cm^2^/m^2^), the correlation between CSMA at C3 and CSMA at L3 was 0.681 (p < 0.01). In the 133 patients with normal SMM (lumbar SMI >43.2cm^2^/m^2^) the correlation was 0.651 (p < 0.01). Using the previously mentioned multivariate prediction formula (29), SMM at L3 was predicted from SMM at C3. In patients with normal SMM, correlation between predicted and measured SMM at L3 was 0.756 (p < 0.01). In patients with low SMM, a correlation between predicted and measured SMM at L3 of 0.751 (p < 0.01) was found. Using the prediction formula and cut-of values of Martin et al. (38), the correlation between estimated and measured SMM at L3 were 0.844 (p <0.01) and 0.864 (p < 0.01) for 98 low and 102 normal SMM patients, respectively (39). Some studies use the lowest (gender specific) quartile as cut-off value for sarcopenia (40). These results highlight the issue that different cut-off values result in different patient numbers being identified as having low SMM. Several cut-off values for low SMI exist, most of which have not been formulated in head and neck cancer patients (29, 38, 41). Very recently, in order to provide standardized cut-off values for low SMM in head and neck cancer patients, Chargi et al. calculated in 1415 HNSCC patients gender and BMI specific cut-off values for low SMM based on mean cervical SMI minus 2 standard deviations. For male patients with BMI < 25 kg/m^2^ and ≥ 25kg/m^2^, a cervical SMI of respectively ≤ 6.8 cm^2^/m^2^ and ≤ 8.5cm^2^/m^2^ was defined for low SMM. For female patients with BMI <25 kg/m^2^ and ≥ 25kg/m^2^, a cervical SMI of respectively ≤ 5.3 cm^2^/m^2^ and ≤ 6.4 cm^2^/m^2^ was defined for low SMM (42).

Although CSMA measurement on CT may be preferred over MRI, because of its more labor-intensive SMM measurement, the latter may be in some patients the only routine cross-sectional imaging available depending on the site of the primary tumor and diagnostic protocols. In quantifying CSMA of the paravertebral muscles and both sternocleidomastoid muscles at the level of C3 on CT and MRI (performed within 1 month of each other) a significant correlation (ICC of 0.97) was found (43). Zwart et al. (44) confirmed this high correlation and found that CT and MRI correlated highly on CSA and SMI (r = 0.958-0.998, p < 0.001). Using the previously defined prediction formula and cut-off value of ≤ 43.2cm^2^/m^2^ for lumbar SMI no significant difference between CT and MRI in diagnosing low SMM was found. Also, for CSMA measurement on MRI an excellent intra-observer agreement was found (ICC 0.961-0.998) (44). Assessment of skeletal muscle CSMA at the level of L3 can thus be assessed using skeletal muscle CSA measurement at the level of C3 on CT or MRI.

Findings above mentioned allow for easy and robust skeletal muscle mass measurements on routinely performed CT and MRI of the head and neck for diagnosis and treatment evaluation. However, CSMA assessment of sternocleidomastoid muscle or paravertebral muscles at cervical levels may be impaired by infiltration of primary tumor or lymph node metastasis into muscles and by previous neck dissection, radical tumor resection with muscle sacrifice or radiotherapy. Although Swartz et al. proposed doubling the area of the SCM that could be measured when SCM measurement is impaired by unilateral lymph node metastases (28), alternative SMM measurement methods have been investigated.

#### Assessment of Cross-Sectional Skeletal Muscle Area at the Level of Other Vertebrae

Vangelov et al. systematically reviewed alternative vertebral levels for SMM evaluation when CT slices at level L3 are not available. Other vertebral landmarks like thoracic vertebrae (Th) Th2, Th4, Th5, Th8, Th10, Th12 and L1 have been used in cancer patients (45). Most of these levels are not included on a routinely performed CT of the head and neck and are not validated against L3 or whole-body CT or MRI. Matsuyama et al. developed a formula (including age, sex, and weight) to estimate the L3 level CSMA using the Th12 level CSMA on chest CT in 164 oral squamous cell carcinoma patients. Correlations between the predicted and measured L3 level CSA were excellent (r = 0.915 and ICC = 0.911) (46). Van Heusden et al. investigated the correlation between SMM measurements at the level of Th4 and L3 in 47 trauma patients and 194 head and neck cancer patients. CSMA at level Th4 strongly correlates with L3 CSMA (r = 0.791). A multivariate model (prediction formula) incorporating the patient characteristics arm positioning (downwards or upwards), age, sex, and weight achieved an even stronger correlation (r = 0.856) (47). It can be concluded that CSMA measured at these levels is a feasible alternative to measurements at L3, particularly when assessment at level C3 is not possible.

Interestingly, Choi et al. did not measure SMM using the CSMA at one axial slide, but a volumetric measurement of the area from hyoid bone anteriorly to third cervical vertebrae posteriorly and caudally to the level of the first rib, immediately above the apical lungs, in 79 HNC patients before and after radiotherapy (mean interscan interval 8.7 ± 5.3 months). Cervical skeletal muscle volume and other volumetric body composition changes were associated with overall survival (48).

Yunaiyama et al. compared an infrahyoid SMI (contouring cross-sectionally paravertebral and sternocleidomastoid muscles) with SMI at level L3 and found a moderate correlation (r = 0.434). No detailed information on the precise infrahyoid level was reported (49).

#### Assessment of Cross-Sectional Skeletal Muscle Area of Masticatory Muscles

As an alternative to CSMA at vertebral levels, masticatory-skeletal muscle (pterygoid and masseter muscles) index assessed at the mandibular notch level has been introduced. Chang et al. (50) recently demonstrated a strong association between the masticatory-skeletal muscle index and SMI at level L3 in 50 trauma and 52 HNC patients (r = 0.901). They stated that masticatory-skeletal muscle index assessment by head and neck CT evaluation is less susceptible to interference by lymphadenopathy and muscle ingrowth by tumor, and differences in measurement methods, and may be readily used as a marker of systemic SMM in patients (50). More extensively, masseter muscle characteristics (masseter volume, masseter skeletal muscle area and masseter thickness) and their relationship with L3 and C3 CSMA were analyzed by Van Heusden et al. (51) in 99 HNC patients. Moderate to strong correlations between the masseter muscle volume (r = 0.531 and 0.699), masseter CSMA (r=0.451 and 0.586) and masseter thickness (r = 0.431 and 0.509) with C3 CSMA and L3 CSMA were found. In patients without cross-sectional imaging at level L3 or C3 or with impaired C3 measurements, masseter muscle parameters could serve as an alternative for SMM assessed by CSMA measurements at these vertebral levels. However, dental status may impact masseter function and size, and (dental) implants may cause scattering hampering reliable masseter measurements on CT (38). Also temporalis muscle thickness has been used as an indicator of sarcopenia. In patients with brain metastases from lung cancer or melanoma a strong association (r= 0.733) between temporal muscle thickness and lumbar skeletal muscle cross-sectional area was found (52). However, no studies have been reported to confirm this correlation in HNC patients. In the only study on temporal muscle thickness as a surrogate parameter for pre-treatment sarcopenia in HNC patients, temporal muscle thickness could predict progression free survival. In this study a high correlation (ICC of 0.894) between of temporal muscle thickness on CT and MRI was found (53).

#### Assessment of Cross-Sectional Skeletal Muscle Area on Extremities

Ultrasound has been suggested to be a quick, cheap, repeatable alternative for SMM measurements on CT or MRI. The SARCUS working group published recommendations for standardization of the use of ultrasound to assess muscle. Recommendations were made for patient positioning, system settings and components to be measured. Standardized anatomical landmarks and measuring points were proposed for different muscles/muscle groups. Muscle parameters included muscle thickness, cross-section area, muscle volume, pennation angle, fascicle length, echo-intensity, stiffness, contraction potential and microcirculation (54, 55). In a systematic review Van den Broeck et al. found that ultrasound-derived equations to estimate SMM are valid and applicable in a healthy population. They advise the clinician to choose an equation that best matches the population the equation was developed for (56). In a systematic review of 17 studies Nijholt et al. (57) showed that ultrasound is a reliable and valid tool to quantify muscles in older (≥ 60 years) adults. Muscle thickness, CSMA and muscle volume were assessed of different muscles among which vastus lateralis, rectus femoris and anterior surface of the upper arm were the most reliable. Two studies describing the validity of ultrasound to predict lean body mass showed good validity as compared with DEXA (r^2^ = 0.92 to 0.96) (57). Galli et al. found that CSMA of the rectus femoris muscle measured by ultrasound was a reliable method for identification of patients with low SMM in a cohort of 47 surgically treated advanced stage HNSCC patients, defining a subset at high-risk of 30-day complications and poorer OS (58). In an expanded cohort of 65 HNC patients, CSMA of rectus femoris muscle measured by ultrasound and CSMA of paravertebral muscles and sternocleidomastoid muscles at level C3 measured on CT and MRI were both independent predictive factors for 30-day major postoperative complications (OR 7.07, p = 0.004 and OR 6.74, p = 0.005, respectively (59).

#### Bioelectrical Impedance Analysis

Bioelectrical impedance analysis (BIA) can also be used to detect low SMM in clinical practice (60, 61). BIA is based on the difference in electrical conductance of tissues; muscle has a high water content and therefore low electrical resistance, whereas fat has a lower water content and higher resistance. Inherently, BIA results are confounded by alterations in hydration status and rapid weight change, for example in patients with edema, dehydration and/or malnutrition, often present in HNC patients. Measurements obtained from routine clinical imaging may be an easily available and more practical approach without extra costs and radiation exposure. However, an advantage of BIA maybe the possibility to perform more easily serial measurements (62). In a prospective observational study of 50 HNC patients undergoing radiotherapy baseline bioelectrical impedance analysis (BIA) measures of skeletal muscle mass, fat-free mass, and fat mass were compared to CT-based assessments. BIA measures of body composition were strongly correlated with CT measures (r = 0.95-0.97) (63).

It can be concluded that there are many methods used to assess SMM in HNC patients of which CSMA measurement at level C3 on CT is the most frequently used and highly correlated alternative for CSMA measurement at level L3 (**Table 1**).

**Table 1:** Different skeletal muscle mass measurement methods and their correlation with skeletal muscle mass measurement on CT at level L3 and C3.

Modality	Level	Muscles	Measure	CT L3	CT C3
CT		Pterygoid + masseter muscles	CSMA	r = 0.901 (50)	
		Masseter muscles	CSMA volume thickness	r = 0.451 (51)	r = 0.586 (51)
				r = 0.531 (51)	r = 0.699 (51)
				r = 0.431 (51)	r = 0.509 (51)
		Temporalis muscles	Thickness	NA (52)	
	C2	Paravertebral muscles		r = 0.810 (35)	
	C3	Paravertebral + sternocleidomastoid muscles	CSMA	r = 0.785 (28)	
				r = 0.75 (30)	
				r^2^ = 0.421 (33)	
				r= 0.810 (34)	
			CSMA multivariate formula	r = 0.891 (28)	
				r = 0.82 (30)	
				r^2^ = 0.721 (33)	
				r = 0.975 (34)	
		Paravertebral muscles	CSMA	r = 0.778 (28)	
				r = 0.877 (35)	
				r = 0.876 (37)*	
				r = 0.381 (37)**	
		Sternocleidomastoid muscles	CSMA	r=0.546 (36)	
			CSMA multivariate formula	r = 0.929 (37)*	
				r = 0.763 (37)**	
	C4	Paravertebral muscles		r = 0.827 (35)	
	Infrahyoid	Paravertebral + sternocleidomastoid muscles	CSMA	r = 0.434 (49)	
	Th4	Pectoralis (minor and major), erector spinae, levator scapulae, rhomboid (minor and major) +transversospinalis muscles	CSMA	r = 0.791 (47)	
			CSMA multivariate formula	r = 0.856 (47)	
	Th12		CSMA	r = 0.915 (46)	
MRI	C3	Paravertebral + sternocleidomastoid muscles	CSMA		r^2^ = 0.94 (43)
					r = 0.958 (44)
ultrasound		Rectus femoris muscles	CSMA	NA (57, 58)	
BIA			CSMA multivariate formula	r = 0.97 (63)	

*Non-sarcopenic patients; **sarcopenic patients.

CSMA, cross-sectional muscle area; NA, not available.

### Muscle Function Measurements

The EWGSOP recommended to focus on low muscle strength as a key characteristic of sarcopenia, to use detection of low muscle quantity and quality to confirm the sarcopenia diagnosis, and to identify poor physical performance as indicative of severe sarcopenia. To assess for evidence of sarcopenia, EWGSOP recommended use of grip strength or a chair stand measure with specific cut-off-points for each test. Measuring grip strength is simple and inexpensive and therefore suitable in daily clinical practice as surrogate for more complicated measures of strength in other body compartments, e.g. arm and leg strength. To diagnose sarcopenia hand grip strength values should be adjusted for sex and BMI (11). The chair stand test (also called chair rise test) can be used as a proxy for strength of leg muscles (quadriceps muscle group). The chair stand test measures the amount of time needed for a patient to rise five times from a seated position without using the arms. It can also be measured as how many times a patient can rise and sit in the chair over a 30-second interval (11).

As function measurements require prospective study design, the number of studies analyzing muscle strength are significantly less than studies on SMM measurements, which can be performed on retrospective cohorts with available scans. Douma et al. (64) investigated muscle function in 254 newly diagnosed HNC patients. Older patients, females, patients with more comorbidity, patients with a normal weight (compared to patients with overweight and obesity), patients living alone and surpisingly patients with no history of smoking (compared to patients with a history of smoking) had significant lower handgrip strength. Older patients, patients with a low educational level, smokers and patients with more comorbidities had a significant lower chair-stand test (64).

Kowshik et al. investigated in 22 HNC patients the association of skeletal muscle function with body composition in HNC patients. Decreased handgrip strength and endurance had a significant but moderate correlation (r = 0.757) with low SMM (65). Only a few studies used the combination of muscle function and skeletal muscle mass for the prediction of treatment outcome in HNC patients (66–69).

### Adverse Events and Prognosis

Due to heterogeneity in HNC patients, research is necessary to understand what, and if any, relationship exists between sarcopenia and treatment outcomes. This knowledge may help in individualizing treatment goals which aims at structural and functional preservation, amelioration of treatment outcomes and the maintenance of quality of life. Sarcopenia is increasingly recognized for its predictive value for treatment-related adverse events in cancer patients. Sarcopenic HNC patients are more likely to experience moderate to severe toxicities of chemoradiation (70, 71). SMM may explain the heterogeneity of patient’s tolerance for chemotherapy to some extent and several studies have shown the predictive value of low SMM on dose limiting toxicity (DLT) in HNC patients treated by radiation and concurrent cisplatin (29, 72–76). A low SMM appeared to be a significant predictor of non-completion of concurrent cisplatin-based chemoradiation. Patients with a low SMM experienced DLT from cisplatin three times more often than patients with a normal SMM (29, 74, 76). Patients experiencing cisplatin DLT had a significant lower survival than patients who did not (29, 74–76). This increased DLT in patients with low SMM may be partly explained by accumulation of cisplatin in muscles and other tissues.

In a systematic review of 3,461 HNC patients a pretreatment low SMM was independently associated with prolonged radiotherapy breaks and chemotherapy-related toxicities (77). Low SMM is a predictor for the risk of aspiration pneumonia in HNSCC patients receiving chemoradiation (78). Moreover, low SMM was found to be predictive for length of hospital stay and unplanned admission in HNC patients treated with (chemo)radiotherapy (79). Low SMM was also associated with long-term morbidity of (chemo)radiation like dysphagia, xerostomia and trismus (40, 80, 81).

Also, in surgically treated HNC patients with low SMM or sarcopenia, higher rates of complications have been found that potentially can delay recovery and increase mortality. In a systematic review Surov and Wienke found that sarcopenia was associated with occurrence of severe postoperative complications (82). Low SMM was associated with early complications, e.g. pneumonia, venous thromboembolism, prolonged ventilation, need for blood transfusion, delirium, fistula and wound disruption, and discharge to post–acute care facilities and readmission (83–88). Orzell et al. found that in patients undergoing major head and neck surgery the combination of low SMM and low muscular function was associated with an increased risk of severe complications and increased length of hospital stay, while in patients with solely low SMM they did not observe this (66).

Several studies have reported on a decreased survival of HNC patients with low SMM. Systemic reviews and meta-analyses in HNC patients showed an association of low SMM with disease free survival, disease specific survival and overall survival for different treatments, different tumor sites and different SMM assessments (82, 89–91). Chargi et al. (67) found that in elderly (≥ 70 years) HNSCC patients, sarcopenia, defined as the combination of low SMM and low muscle function (handgrip strength and/or 4-m gait speed), was a better predictor of OS than low SMM or low muscle function only. Of the 85 included patients 81.2% had low SMM, 58.8% had low hand grip strength, 68.2% had low gait speed and 48.2% were classified as sarcopenic. SMM, handgrip strength and gait speed correlated significantly with age (67).

In conclusion, HNC patients with low SMM with or without low muscular function experience more toxicity of cisplatin and radiotherapy, leading to significantly more frequent dose limiting toxicity and radiotherapy breaks, and complications in major head and neck surgery. Low SMM with or without low muscular function is also associated with decreased survival. Therefore, low SMM seems suitable to be used for more individualized (alternative) treatment planning in head and neck cancer patients.

## Frailty

Frailty is often defined as an age-related cumulative decline across multiple physiologic systems, with impaired homeostatic reserve and a reduced capacity of the organism to withstand stress, leading to increased risk of adverse health outcomes. In HNC frailty is associated with severe chemoradiation-related complications (92), postoperative complications (93, 94), life-threatening postoperative complications requiring intensive care unit (ICU) admission (95), length of hospital stay and unplanned readmission (96), discharge to short-term or skilled nursing facilities (94), 30-day mortality after head and neck oncologic surgery (95), poor survival after chemoradiation (92) and decline in health-related quality of life after treatment (97). A comprehensive geriatric assessment (CGA) which evaluates physical, psychological, functional, and social capabilities and limitations of geriatric patients is the gold standard to diagnose frailty. In geriatric oncology, CGA is used to detect disabilities and comorbid conditions that potentially contribute to an older patient’s vulnerability, predisposing to poor outcome and treatment complications, so that treatment can be adjusted accordingly. However, such assessments are time-consuming, leading cancer specialists to seek a short screening tool that can separate fit older patient with cancer, who are able to receive standard cancer treatment, from vulnerable patients that should subsequently receive a full assessment to guide tailoring of their treatment regimen. Among the many screening instruments, which are used to predict the presence of impairments on CGA in elderly patients with cancer, are the Vulnerable Elders Survey-13 (VES-13), Geriatric 8 (G8), Triage Risk Screening Tool (TRST), Groningen Frailty Index (GFI), Fried frailty criteria, Barber and abbreviated CGA (aCGA) (98–100). The G8 screening tool, developed specifically for older patient with cancer, has the highest sensitivity, which is important to select patients who may benefit from CGA. Unfortunately, the specificity and negative predictive value are limited (98–100). This frailty screening tool consists of eight items which cover multiple geriatric domains, including nutritional status, physical capacity, mood, and polypharmacy. Scores range from zero to seventeen, with scores ≤ fourteen representing potential frailty (101).

## Sarcopenia and Frailty

Sarcopenia is a major component of frailty. Although sarcopenia can lead to frailty, not all patients with sarcopenia are frail. In fact, sarcopenia is about twice as common as frailty (14).

### Association of Sarcopenia and Frailty

Williams et al. (102) investigated the association of single-slice CT-assessed muscle measurements at the level of L3 with the 36-item Carolina Frailty Index in 162 older (>65 years) adults with cancer. Muscle measurements included SMI, skeletal muscle density (SMD, average Hounsfield Units of CSMA) and their multiplication skeletal muscle gauge (SMG=SMI x SMD). SMG can thus be regarded as a combination of muscle quantity and quality. The association between Carolina Frailty Index and SMI was not significant, and the correlation was weak (r = -0.08). For SMD and SMG this association was significant, but correlations were moderate (r = -0.33 and -0.30, respectively) (103). Dunne et al. (103) investigated in 100 geriatric oncologic patients the association of sarcopenia, defined as SMM measured at the level of third lumbar vertebra only, and several metrics of CGA. No significant association between SMM and instrumental activities of daily living (IADL), falls, lower short physical performance battery score, depression, fatigue, or self-reported exercise were found (103). In these studies, HNC patients were not (separately) analyzed.

Zwart et al. (32) were the first to demonstrate that low SMM is independently associated with frailty in HNC patients. In 112 advanced stage HNC patients SMM was assessed on CT at the level of C3. Frailty was evaluated by Geriatrics 8 (G8), Groningen Frailty Indicator, Timed Up and Go test, and Malnutrition Universal Screening Tool. SMI correlated best with the G8 score (r = 0.38, p < 0.001), followed by the GFI score (r = −0.27, p = 0.004). Timed Up and Go and SMI did not correlate significantly with each other (r = −0.11). The G8 score was found to be an independent variable associated with SMI (OR 0.76, 95% CI 0.66–0.89, p < 0.001) (85). Meerkerk et al. (68) confirmed this finding in 150 HNC patients (≥ 60-years old) and they found a weak correlation between G8 frailty score and SMI (r = 0.252, p < 0.01), but not when combined with handgrip strength. SMI was an independent variable associated with G8 (OR 0.92, 95% CI 0.86–0.98, p = 0.006). Handgrip strength itself showed also a significant but weak correlation with the G8 frailty score (r = 0.284, p < 0.01) (68). In a sequel study, the association between sarcopenia and frailty was investigated in 73 elderly (≥ 70-years) HNSCC patients. Sarcopenia was defined as the combination of reduced handgrip strength and low skeletal muscle mass (assessed on CT at level C3), according to the EWGSOP-2 criteria. Frailty screening was performed using the GFI and the Fried criteria and a CGA by a geriatrician. Low SMI was the only significant predictor for frailty diagnosed by CGA, independent of comorbidity and muscle strength (105). From these studies it can be concluded that low SMI may have potential to predict frailty and is a promising time-efficient and routinely available tool for clinical practice.

### Comparing the Predictive Role of Sarcopenia and Frailty

Galli et al. (58) investigated the potential role low SMM as assessed by ultrasound of the rectus femoris muscle in a group of patients with locally advanced HNC that underwent surgery and compared its predictive role with more commonly employed clinical predictors of postoperative complications and poor survival. On univariate analysis, ASA score, modified Frailty index and sex-adjusted rectus femoris CSMA were found to be statistically significant predictors of 30-day postoperative complications. At multivariate analysis, with a model including ASA score, modified Frailty index and CSMA, only sex-adjusted rectus femoris CMSA was confirmed as significant predictor of 30-day complications (OR 9.84, 95% CI 2.11–45.77). On univariate analysis, ASA score, Charlson comorbidity index, modified Frailty index and sex-adjusted rectus femoris CSMA were found to be statistically significant predictors of overall survival. At multivariate analysis, with a model including ASA score, Charlson comorbidity index, modified Frailty index and CSMA, only rectus femoris CMSA was confirmed as significant predictor of overall survival (OR 4.42, 95% CI 1.12–17.40; p = 0.033). Low SMM as assessed by ultrasound measurement of CSMA of the rectus femoris muscle was an independent and stronger predictor of complications and survival than the modified Frailty index (58).

Mascarella et al. (104) investigated predictive factors for postoperative adverse events in 127 treatment-naïve HNC patients undergoing surgery with microvascular reconstruction. For SMM assessment CSMA of the paravertebral muscles at C3 or L3 was measured on CT. Low SMI was independently associated with severe complications (Clavien-Dindo Grade 3+ events; OR 2.80, 95% CI 1.18–6.99), particularly fistula (OR 6.10, 95% CI of 1.53–24.3), when adjusted for multiple factors. SMI outperformed the modified Frailty index and preoperative anaesthesia risk assessment index to predict postoperative adverse events: for the prediction of Clavien-Dindo grade 3+ complications the areas under the curve for SMI, modified Frailty index and preoperative anaesthesia risk assessment index were 0.76, 0.56 and 0.50 (p < .05), respectively. The overall accuracy of the SMI to predict a Clavien-Dindo grade 3+ complication, fistula, or prolonged stay in hospital was 84.8% (104).

### Sarcopenia and Frailty as Entities

Although sarcopenia and frailty have some commonalities and are often used interchangeably, they appear to represent separate entities with different constructs. Sarcopenia is characterized by loss of skeletal muscle and function, while frailty is a broader term used to indicate reduced homeostatic reserves. The most apparent overlap of sarcopenia and frailty is impaired physical function and disability. Both sarcopenia and frailty are highly prevalent age-related conditions that are associated with adverse outcomes. There is growing consensus that although sarcopenia may be a component of frailty, frailty is more multifaceted than sarcopenia alone. The general concept of frailty goes beyond physical factors and encompasses social and psychological dimensions as well, including social support and cognitive function. Furthermore, therapeutic approaches to the two age-related conditions may also vary. Treatment of sarcopenia is focused on combining exercise and adequate protein intake to increasing muscle mass and strength, while frailty is focused on a broader set of physical and non-physical domains (102).

Although discordance between frailty and low SMM has been reported more often, comparing studies is difficult, as a variety of definitions are employed. Studies using a physical frailty definition tend to show more overlap with sarcopenia, because it uses low muscle function, e.g. handgrip strength, as one of the criteria. Similarly, sarcopenia definitions that include reduced muscle function, e.g. handgrip strength and low performance, e.g. gait speed, have more concordance with frailty. While there is some overlap between sarcopenia and frailty, the consensus is that they are distinct (102).

Nevertheless, both sarcopenia and frailty can predict adverse outcomes and can be used to identify vulnerable patients, select treatment options, adjust treatments, improve patient counselling, improve preoperative nutritional status and anticipate early on complications, length of hospital stay and discharge. Depending on their usefulness in clinical practice one condition or both of these conditions can be used to individualize treatment in clinical practice.

## Conclusions

Sarcopenia is proposed to be a combination of low muscle quantity and muscle function. However, in most studies on sarcopenia in patients with HNC only SMM is assessed. There are many methods to measure SMM, but the most often used methods are to measure CSMA on CT or MRI at the level of L3 or C3. Many different cut-off values for these SMM parameters to define low SMM have been used. Also, to diagnose frailty many instruments and definitions have been used in HNC patients. The association between sarcopenia and frailty depends on definitions, measurement methods and cut-off values used. Nevertheless, both can predict adverse outcomes and can be used to tailor treatments. It has to be decided which condition is most predictive and clinically useful in assessing older HNC patients and treatment decision making. More research is needed to investigate if sarcopenia and frailty, depending on the definitions and measurements used, can be used interchangeably, or have additional value and should be used in combination to optimize individualized treatment.

## Author’s Note

This article was written by members and invitees of the International Head and Neck Scientific Group (http://www.IHNSG.com).

## Author Contributions

RB and CM contributed to conception and design of the study. RB wrote the first draft of the manuscript. All authors contributed to manuscript revision, read, and approved the submitted version.

## Conflict of Interest

The authors declare that the research was conducted in the absence of any commercial or financial relationships that could be construed as a potential conflict of interest.

## Publisher’s Note

All claims expressed in this article are solely those of the authors and do not necessarily represent those of their affiliated organizations, or those of the publisher, the editors and the reviewers. Any product that may be evaluated in this article, or claim that may be made by its manufacturer, is not guaranteed or endorsed by the publisher.

## References

[1] Rettig EM D'Souza G. Epidemiology of Head and Neck Cancer. Surg Oncol Clin N Am (2015) 24(3):379–96. doi: 10.1016/j.soc.2015.03.001 25979389

[2] Economopoulou P de Bree R Kotsantis I Psyrri A. Diagnostic Tumor Markers in Head and Neck Squamous Cell Carcinoma (HNSCC) in the Clinical Setting. Front Oncol (2019) 9:827. doi: 10.3389/fonc.2019.00827 31555588 PMC6727245

[3] Cervenka BP Rao S Bewley AF. Head and Neck Cancer and the Elderly Patient. Otolaryngol Clin North Am (2018) 51(4):741–51. doi: 10.1016/j.otc.2018.03.004 29801920

[4] Teymoortash A Ferlito A Halmos GB. Treatment in Elderly Patients With Head and Neck Cancer : A Challenging Dilemma. HNO (2016) 64(4):217–20. doi: 10.1007/s00106-016-0138-6 26992385

[5] Coca-Pelaz A Halmos GB Strojan P de Bree R Bossi P Bradford CR. The Role of Age in Treatment-Related Adverse Events in Patients With Head and Neck Cancer: A Systematic Review. Head Neck (2019) 41(7):2410–29. doi: 10.1002/hed.25696 30737976

[6] Teymoortash A Halmos GB Silver CE Strojan P Haigentz M Jr Rinaldo A. On the Need for Comprehensive Assessment of Impact of Comorbidity in Elderly Patients With Head and Neck Cancer. Eur Arch Otorhinolaryngol (2014) 271(10):2597–600. doi: 10.1007/s00405-014-3203-3 25060978

[7] Prado CM Cushen SJ Orsso CE Ryan AM. Sarcopenia and Cachexia in the Era of Obesity: Clinical and Nutritional Impact. Proc Nutr Soc (2016) 75(2):188–98. doi: 10.1017/S0029665115004279 26743210

[8] Rosenberg IH. Sarcopenia: Origins and Clinical relevance J Nutr (1997) 127(5 Suppl):990S–1S. doi: 10.1093/jn/127.5.990S 9164280

[9] Biolo G Cederholm T Muscaritoli M. Muscle Contractile and Metabolic Dysfunction is a Common Feature of Sarcopenia of Aging and Chronic Diseases: From Sarcopenic Obesity to Cachexia. Clin Nutr (2014) 33:737–48. doi: 10.1016/j.clnu.2014.03.007 24785098

[10] Cruz-Jentoft AJ Baeyens JP Bauer JM Boirie Y Cederholm T Landi F. Sarcopenia: European Consensus on Definition and Diagnosis: Report of the European Working Group on Sarcopenia in Older People. Age Ageing (2010) 39(4):412–23. doi: 10.1093/ageing/afq034 PMC2886201 20392703

[11] Cruz-Jentoft AJ Bahat G Bauer J Boirie Y Bruyère O Cederholm T. Sarcopenia: Revised European Consensus on Definition and Diagnosis. Age Ageing (2019) 48(1):16–31. doi: 10.1093/ageing/afy169 30312372 PMC6322506

[12] Bhasin S Travison TG Manini TM Patel S Pencina KM Fielding RA. Sarcopenia Definition: The Position Statements of the Sarcopenia Definition and Outcomes Consortium. J Am Geriatr Soc (2020) 68:1410–8. doi: 10.1111/jgs.16372 PMC12132920 32150289

[13] Muscaritoli M Anker SD Argilés J Aversa Z Bauer JM Biolo G. Consensus Definition of Sarcopenia, Cachexia and Pre-Cachexia: Joint Document Elaborated by Special Interest Groups (SIG) “Cachexia-Anorexia in Chronic Wasting Diseases” and “Nutrition in Geriatrics”. Clin Nutr (2010) 29(2):154–9. doi: 10.1016/j.clnu.2009.12.004 20060626

[14] von Haehling S Morley JE Anker SD. An Overview of Sarcopenia: Facts and Numbers on Prevalence and Clinical Impact. J Cachexia Sarcopenia Muscle (2010) 1(2):129–33. doi: 10.1007/s13539-010-0014-2 PMC3060646 21475695

[15] Christensen JF Jones LW Andersen JL Daugaard G Rorth M Hojman P. Muscle Dysfunction in Cancer Patients. Ann Oncol (2014) 25:947–58. doi: 10.1093/annonc/mdt551 24401927

[16] Hébuterne X Lemarié E Michallet M de Montreuil CB Schneider SM Goldwasser F. Prevalence of Malnutrition and Current Use of Nutrition Support in Patients With Cancer. J Parenter Enter Nutr (2014) 38(2):196–204. doi: 10.1177/0148607113502674 24748626

[17] Silva PB Ramos GHA Petterle RR Borba VZC. Sarcopenia as an Early Complication of Patients With Head and Neck Cancer With Dysphagia. Eur J Cancer Care (Engl) (2021) 30(1):e13343. doi: 10.1111/ecc.13343 33043532

[18] Chen KC Jeng Y Wu WT Wang TG Han DS Özçakar L. Sarcopenic Dysphagia: A Narrative Review From Diagnosis to Intervention. Nutrients (2021) 13(11):4043. doi: 10.3390/nu13114043 34836299 PMC8621579

[19] Mascarella MA Patel T Vendra V Gardiner L Kergoat MJ Kubik MW. Poor Treatment Tolerance in Head and Neck Cancer Patients With Low Muscle Mass. Head Neck (2022). doi: 10.21037/qims-21-911 PMC11412609 35020252

[20] Albano D Messina C Vitale J Sconfienza LM. Imaging of Sarcopenia: Old Evidence and New Insights. Eur Radiol (2020) 30(4):2199–208. doi: 10.1007/s00330-019-06573-2 31834509

[21] Prado CM Heymsfield SB. Lean Tissue Imaging. J Parenter Enter Nutr (2014) 38(8):940–53. doi: 10.1177/0148607114550189 PMC4361695 25239112

[22] Lee SY Gallagher D. Assessment Methods in Human Body Composition. Curr Opin Clin Nutr Metab Care (2008) 11(5):566–72. doi: 10.1097/MCO.0b013e32830b5f23 PMC2741386 18685451

[23] Cespedes Feliciano EM Popuri K Cobzas D Baracos VE Beg MF Khan AD. Evaluation of Automated Computed Tomography Segmentation to Assess Body Composition and Mortality Associations in Cancer Patients. J Cachexia Sarcopenia Muscle (2020) 11(5):1258–69. doi: 10.1002/jcsm.12573 PMC7567141 32314543

[24] Lee YS Hong N Witanto JN Choi YR Park J Decazes P. Deep Neural Network for Automatic Volumetric Segmentation of Whole-Body CT Images for Body Composition Assessment. Clin Nutr (2021) 40(8):5038–46. doi: 10.1016/j.clnu.2021.06.025 34365038

[25] Shen W Punyanitya M Wang Z Gallagher D St-Onge MP Albu J. Total Body Skeletal Muscle and Adipose Tissue Volumes: Estimation From a Single Abdominal Cross-Sectional Image. J Appl Physiol (2004) 97(6):2333–8. doi: 10.1152/japplphysiol.00744.2004 15310748

[26] Heymsfield SB Wang Z Baumgartner RN Ross R. Human Body Composition: Advances in Models and Methods. Annu Rev Nutr (1997) 17(1):527–58. doi: 10.1146/annurev.nutr.17.1.527 9240939

[27] Prado CM Lieffers JR McCargar LJ Reiman T Sawyer MB Martin L. Prevalence and Clinical Implications of Sarcopenic Obesity in Patients With Solid Tumours of the Respiratory and Gastrointestinal Tracts: A Population-Based Study. Lancet Oncol (2008) 9(7):629–35. doi: 10.1016/S1470-2045(08)70153-0 18539529

[28] Swartz JE Pothen AJ Wegner I Smid EJ Swart KM de Bree R. Feasibility of Using Head and Neck CT Imaging to Assess Skeletal Muscle Mass in Head and Neck Cancer Patients. Oral Oncol (2016) 62:28–33. doi: 10.1016/j.oraloncology.2016.09.006 27865369

[29] Wendrich AW Swartz JE Bril SI Wegner I de Graeff A Smid EJ. Low Skeletal Muscle Mass is a Predictive Factor for Chemotherapy Dose-Limiting Toxicity in Patients With Locally Advanced Head and Neck Cancer. Oral Oncol (2017) 71:26–33. doi: 10.1016/j.oraloncology.2017.05.012 28688687

[30] Bril SI Chargi N Pezier TF Tijink BM Braunius WW Smid EJ. Validation of Skeletal Muscle Mass Assessment at the Level of the Third Cervical Vertebra in Patients With Head and Neck Cancer. Head Neck (2022) 44(2):307–16. doi: 10.1002/hed.26927

[31] Bril SI Wendrich AW Swartz JE Wegner I Pameijer F Smid EJ. Interobserver Agreement of Skeletal Muscle Mass Measurement on Head and Neck CT Imaging at the Level of the Third Cervical Vertebra. Eur Arch Otorhinolaryngol (2019) 276(4):1175–82. doi: 10.1007/s00405-019-05307-w PMC6426814 30689037

[32] Zwart AT van der Hoorn A van Ooijen PMA Steenbakkers RJHM de Bock GH Halmos GB. CT-Measured Skeletal Muscle Mass Used to Assess Frailty in Patients With Head and Neck Cancer. J Cachexia Sarcopenia Muscle (2019) 10(5):1060–9. doi: 10.1002/jcsm.12443 PMC6818448 31134765

[33] Jung AR Roh JL Kim JS Choi SH Nam SY Kim SY. Efficacy of Head and Neck Computed Tomography for Skeletal Muscle Mass Estimation in Patients With Head and Neck Cancer. Oral Oncol (2019) 95:95–9. doi: 10.1016/j.oraloncology.2019.06.009 31345401

[34] Lu X Tian Y Huang J Li F Shao T Huang G. Evaluating the Prognosis of Oral Squamous Cell Carcinoma Patients *via* L3 Skeletal Muscle Index. Oral Dis (2021) 12. doi: 10.1111/odi.14074 34773352

[35] Ufuk F Herek D Yüksel D. Diagnosis of Sarcopenia in Head and Neck Computed Tomography: Cervical Muscle Mass as a Strong Indicator of Sarcopenia. Clin Exp Otorhinolaryngol (2019) 12(3):317–24. doi: 10.21053/ceo.2018.01613 PMC6635710 30947498

[36] Yoshimura T Suzuki H Takayama H Higashi S Hirano Y Tezuka M. Prognostic Role of Preoperative Sarcopenia Evaluation of Cervical Muscles With Long-Term Outcomes of Patients With Oral Squamous Cell Carcinoma. Cancers (Basel) (2021) 13(18):4725. doi: 10.3390/cancers13184725 34572952 PMC8465585

[37] Yoon J-K Jang JY An Y-S Lee SJ. Skeletal Muscle Mass at C3 may Not be a Strong Predictor for Skeletal Muscle Mass at L3 in Sarcopenic Patients With Head and Neck Cancer. PloS One (2021) 16(7):e0254844. doi: 10.1371/journal.pone.0254844 34280248 PMC8289025

[38] Martin L Birdsell L Macdonald N Reiman T Clandinin MT McCargar LJ. Cancer Cachexia in the Age of Obesity: Skeletal Muscle Depletion is a Powerful Prognostic Factor, Independent of Body Mass Index. J Clin Oncol Off J Am Soc Clin Oncol (2013) 31(12):1539–47. doi: 10.1200/JCO.2012.45.2722 23530101

[39] Bril SI van Beers MA Chargi N Carrillo Minulina N Smid EJ Dankbaar JW. Skeletal Muscle Mass at C3 is a Strong Predictor for Skeletal Muscle Mass at L3 in Sarcopenic and non-Sarcopenic Patients With Head and Neck Cancer. Oral Oncol (2021) 122:105558. doi: 10.1016/j.oraloncology.2021.105558 34627078

[40] van Rijn-Dekker MI van den Bosch L van den Hoek JGM Bijl HP van Aken ESM van der Hoorn A. Impact of Sarcopenia on Survival and Late Toxicity in Head and Neck Cancer Patients Treated With Radiotherapy. Radiother Oncol (2020) 147:103–10. doi: 10.1016/j.radonc.2020.03.014 32251949

[41] Zhuang CL Huang DD Pang WY Zhou CJ Wang SL Lou N. Sarcopenia is an Independent Predictor of Severe Postoperative Complications and Long-Term Survival After Radical Gastrectomy for Gastric Cancer. Med (Baltimore) (2016) 95(13):e3164. doi: 10.1097/MD.0000000000003164 PMC4998538 27043677

[42] Chargi N Bril SI Smid EJ de Jong PA de Bree R. Cut-Off Values for Low Skeletal Muscle Mass at the Level of the Third Cervical Vertebra (C3) in Patients With Head and Neck Cancer. Quant Imaging Med Surg (2022) 95(13):e3164. doi: 10.21037/qims-21-911 PMC9131345 35655816

[43] Chargi N Ansari E Huiskamp LFJ Bol G de Bree R. Agreement Between Skeletal Muscle Mass Measurements Using Computed Tomography Imaging and Magnetic Resonance Imaging in Head and Neck Cancer Patients. Oral Oncol (2019) 99:104341. doi: 10.1016/j.oraloncology.2019.06.022 31253538

[44] Zwart AT Becker JN Lamers MJ Dierckx RAJO de Bock GH Halmos GB. Skeletal Muscle Mass and Sarcopenia can be Determined With 1.5-T and 3-T Neck MRI Scans, in the Event That No Neck CT Scan is Performed. Eur Radiol (2021) 31(6):4053–62. doi: 10.1007/s00330-020-07440-1 PMC8128750 33219847

[45] Vangelov B Bauer J Kotevski D Smee RI. The Use of Alternate Vertebral Levels to L3 in Computed Tomography Scans for Skeletal Muscle Mass Evaluation and Sarcopenia Assessment in Patients With Cancer: A Systematic Review. Br J Nutr (2021) 29:1–14. doi: 10.1017/S0007114521001446 33910664

[46] Matsuyama R Maeda K Yamanaka Y Ishida Y Kato R Nonogaki T. Assessing Skeletal Muscle Mass Based on the Cross-Sectional Area of Muscles at the 12th Thoracic Vertebra Level on Computed Tomography in Patients With Oral Squamous Cell Carcinoma. Oral Oncol (2021) 113:105126. doi: 10.1016/j.oraloncology.2020.105126 33388617

[47] van Heusden HC Swartz JE Chargi N de Jong PA van Baal MCPM Wegner I. Feasibility of Assessment of Skeletal Muscle Mass on a Single Cross-Sectional Image at the Level of the Fourth Thoracic Vertebra. Eur J Radiol (2021) 142:109879. doi: 10.1016/j.ejrad.2021.109879 34343845

[48] Choi Y Ahn KJ Jang J Shin NY Jung SL Kim BS. Prognostic Value of Computed Tomography-Based Volumetric Body Composition Analysis in Patients With Head and Neck Cancer: Feasibility Study. Head Neck (2020) 42(9):2614–25. doi: 10.1002/hed.26310 32543090

[49] Yunaiyama D Okubo M Arizono E Tsukahara K Tanigawa M Nagao T. Sarcopenia at the Infrahyoid Level as a Prognostic Factor in Patients With Advanced-Stage non-Virus-Related Head and Neck Carcinoma. Eur Arch Otorhinolaryngol (2021). doi: 10.1007/s00405-021-07147-z 34697649

[50] Chang SW Tsai YH Hsu CM Huang EI Chang GH Tsai MS. Masticatory Muscle Index for Indicating Skeletal Muscle Mass in Patients With Head and Neck Cancer. PloS One (2021) 16:e0251455. doi: 10.1371/journal.pone.0251455 33970954 PMC8109770

[51] van Heusden HC Chargi N Dankbaar JW Smid EJ de Bree R. Masseter Muscle Parameters can Function as an Alternative for Skeletal Muscle Mass Assessments on Cross-Sectional Imaging at Lumbar or Cervical Vertebral Levels. Quant Imaging Med Surg (2022) 12(1):15–27. doi: 10.21037/qims-21-43 34993057 PMC8666780

[52] Leitner J Pelster S Schöpf V Berghoff AS Woitek R Asenbaum U. High Correlation of Temporal Muscle Thickness With Lumbar Skeletal Muscle Cross-Sectional Area in Patients With Brain Metastases. PloS One (2018) 13(11):e0207849. doi: 10.1371/journal.pone.0207849 30496307 PMC6264824

[53] Lee B Bae YJ Jeong WJ Kim H Choi BS Kim JH. Temporalis Muscle Thickness as an Indicator of Sarcopenia Predicts Progression-Free Survival in Head and Neck Squamous Cell Carcinoma. Sci Rep (2021) 11(1):19717. doi: 10.1038/s41598-021-99201-3 34611230 PMC8492642

[54] Perkisas S Bastijns S Baudry S Bauer J Beaudart C Beckwée D. Application of Ultrasound for Muscle Assessment in Sarcopenia: 2020 SARCUS Update. Eur Geriatr Med (2021) 12(1):45–59. doi: 10.1007/s41999-020-00433-9 33387359

[55] Perkisas S Baudry S Bauer J Beckwée D De Cock AM Hobbelen H. Application of Ultrasound for Muscle Assessment in Sarcopenia: Towards Standardized Measurements. Eur Geriatr Med (2018) 9(6):739–57. doi: 10.1007/s41999-018-0104-9 34674473

[56] Van den Broeck J Buzzatti L Jager-Wittenaar H Perkisas S Scafoglieri A. Application of Ultrasound for Muscle Assessment in Sarcopenia: Towards Standardized Measurements. Clin Nutr ESPEN (2021) 46:133–41. doi: 10.1016/j.clnesp.2021.08.012 34857186

[57] Nijholt W Scafoglieri A Jager-Wittenaar H Hobbelen JSM van der Schans CP. The Reliability and Validity of Ultrasound to Quantify Muscles in Older Adults: A Systematic Review. J Cachexia Sarcopenia Muscle (2017) 8(5):702–12. doi: 10.1002/jcsm.12210 PMC5659048 28703496

[58] Galli A Colombo M Carrara G Lira Luce F Paesano PL Giordano L. Low Skeletal Muscle Mass as Predictor of Postoperative Complications and Decreased Overall Survival in Locally Advanced Head and Neck Squamous Cell Carcinoma: The Role of Ultrasound of Rectus Femoris Muscle. Eur Arch Otorhinolaryngol (2020) 277(12):3489–502. doi: 10.1007/s00405-020-06123-3 32535862

[59] Galli A Colombo M Prizio C Carrara G Lira Luce F Paesano PL. Skeletal Muscle Depletion and Major Postoperative Complications in Locally-Advanced Head and Neck Cancer: A Comparison Between Ultrasound of Rectus Femoris Muscle and Neck Cross-Sectional Imaging. Cancers (Basel) (2022) 14(2):347. doi: 10.3390/cancers14020347 35053512 PMC8774237

[60] Gort-van Dijk D Weerink LBM Milovanovic M Haveman JW Hemmer PHJ Dijkstra G. Bioelectrical Impedance Analysis and Mid-Upper Arm Muscle Circumference Can Be Used to Detect Low Muscle Mass in Clinical Practice. Nutrients (2021) 13(7):2350. doi: 10.3390/nu13072350 34371860 PMC8308498

[61] Jager-Wittenaar H Dijkstra PU Earthman CP Krijnen WP Langendijk JA van der Laan BF. Validity of Bioelectrical Impedance Analysis to Assess Fat-Free Mass in Patients With Head and Neck Cancer: An Exploratory Study. Head Neck (2014) 36(4):585–91. doi: 10.1002/hed.23336 23595994

[62] de Bree R Swartz JE Bril S Chargi N Wegner I Smid EJ. Skeletal Muscle Mass Measurements Using Head and Neck CT Imaging in Head and Neck Cancer Patients. Radiother Oncol (2021) 161:72–3. doi: 10.1016/j.radonc.2021.05.029 34107294

[63] Grossberg AJ Rock CD Edwards J Mohamed ASR Ruzensky D Currie A. Bioelectrical Impedance Analysis as a Quantitative Measure of Sarcopenia in Head and Neck Cancer Patients Treated With Radiotherapy. Radiother Oncol (2021) 159:21–7. doi: 10.1016/j.radonc.2021.03.005 PMC8205950 33736997

[64] Douma JAJ Verdonck-de Leeuw IM Leemans CR Jansen F Langendijk JA Baatenburg de Jong RJ. Demographic, Clinical and Lifestyle-Related Correlates of Accelerometer Assessed Physical Activity and Fitness in Newly Diagnosed Patients With Head and Neck Cancer. Acta Oncol (2020) 59(3):342–50. doi: 10.1080/0284186X.2019.1675906 31608747

[65] Kowshik V Velkumary S Sethi P Feula JM Subhashri S Abirami M. Association of Handgrip Strength and Endurance With Body Composition in Head and Neck Cancer Patients. J Family Med Prim Care (2021) 10(2):910–6. doi: 10.4103/jfmpc.jfmpc_1695_20 PMC8138385 34041097

[66] Orzell S Verhaaren BFJ Grewal R Sklar M Irish JC Gilbert R. Evaluation of Sarcopenia in Older Patients Undergoing Head and Neck Cancer Surgery. Laryngoscope (2022) 132(2):356–63. doi: 10.1002/lary.29782 34383321

[67] Chargi N Bril SI Emmelot-Vonk MH de Bree R. Sarcopenia is a Prognostic Factor for Overall Survival in Elderly Patients With Head-and-Neck Cancer. Eur Arch Otorhinolaryngol (2019) 276(5):1475–86. doi: 10.1007/s00405-019-05361-4 PMC6458984 30830300

[68] Meerkerk CDA Chargi N de Jong PA van den Bos F de Bree R. Sarcopenia Measured With Handgrip Strength and Skeletal Muscle Mass to Assess Frailty in Older Patients With Head and Neck Cancer. J Geriatr Oncol (2021) 12(3):434–40. doi: 10.1016/j.jgo.2020.10.002 33067163

[69] Orell-Kotikangas H Österlund P Mäkitie O Saarilahti K Ravasco P Schwab U. Cachexia at Diagnosis is Associated With Poor Survival in Head and Neck Cancer Patients. Acta Otolaryngol (2017) 137(7):778–85. doi: 10.1080/00016489.2016.1277263 28125312

[70] Yamaguchi T Makiguchi T Nakamura H Yamatsu Y Hirai Y Shoda K. Impact of Muscle Volume Loss on Acute Oral Mucositis in Patients Undergoing Concurrent Chemoradiotherapy After Oral Cancer Resection. Int J Oral Maxillofac Surg (2021) 50(9):1195–202. doi: 10.1016/j.ijom.2020.12.005 33414037

[71] Nagpal P Pruthi DS Pandey M Yadav A Singh H. Impact of Sarcopenia in Locally Advanced Head and Neck Cancer Treated With Chemoradiation: An Indian Tertiary Care Hospital Experience. Oral Oncol (2021) 121:105483. doi: 10.1016/j.oraloncology.2021.105483 34403887

[72] Sealy MJ Dechaphunkul T van der Schans CP Krijnen WP Roodenburg JLN Walker J. Low Muscle Mass is Associated With Early Termination of Chemotherapy Related to Toxicity in Patients With Head and Neck Cancer. Clin Nutr (2020) 39:501–9. doi: 10.1016/j.clnu.2019.02.029 30846324

[73] Ganju RG Morse R Hoover A TenNapel M Lominska CE. The Impact of Sarcopenia on Tolerance of Radiation and Outcome in Patients With Head and Neck Cancer Receiving Chemoradiation. Radiother Oncol (2019) 137:117–24. doi: 10.1016/j.radonc.2019.04.023 31085391

[74] Bril SI Al-Mamgani A Chargi N Remeijer P Devriese LA de Boer JP. The Association of Pretreatment Low Skeletal Muscle Mass With Chemotherapy Dose-Limiting Toxicity in Patients With Head and Neck Cancer Undergoing Primary Chemoradiotherapy With High-Dose Cisplatin. Head Neck (2022) 44(1):189–200. doi: 10.1002/hed.26919 34713519 PMC9298001

[75] Huang X Lv LN Zhao Y Li L Zhu XD. Is Skeletal Muscle Loss Associated With Chemoradiotherapy Toxicity in Nasopharyngeal Carcinoma Patients? A Prospective Study. Clin Nutr (2021) 40(1):295–302. doi: 10.1016/j.clnu.2020.05.020 32507513

[76] Chargi N Bashiri F Wendrich AW Smid EJ de Jong PA Huitema ADR. Image-Based Analysis of Skeletal Muscle Mass Predicts Cisplatin Dose-Limiting Toxicity in Patients With Locally Advanced Head and Neck Cancer. Eur Arch Otorhinolaryngol (2022). doi: 10.1007/s00405-021-07229-y 35038029

[77] Findlay M White K Lai M Luo D Bauer JD. The Association Between Computed Tomography Defined Sarcopenia and Outcomes in Adult Patients Undergoing Radiotherapy of Curative Intent for Head and Neck Cancer: A Systematic Review. J Acad Nutr Diet (2020) 120:1330–47. doi: 10.1016/j.jand.2020.03.021 32711854

[78] Endo K Ueno T Hirai N Komori T Nakanishi Y Kondo S. Low Skeletal Muscle Mass Is a Risk Factor for Aspiration Pneumonia During Chemoradiotherapy. Laryngoscope (2021) 131(5):E1524–9. doi: 10.1002/lary.29165 33030229

[79] Findlay M White K Brown C Bauer JD. Nutritional Status and Skeletal Muscle Status in Patients With Head and Neck Cancer: Impact on Outcomes. J Cachexia Sarcopenia Muscle (2021) 12(6):2187–98. doi: 10.1002/jcsm.12829 PMC8718020 34676673

[80] Lee J Liu SH Chen JC Leu YS Liu CJ Chen YJ. Progressive Muscle Loss is an Independent Predictor for Survival in Locally Advanced Oral Cavity Cancer: A Longitudinal Study. Radiother Oncol (2021) 158:83–9. doi: 10.1016/j.radonc.2021.02.014 33621588

[81] Karsten RT Chargi N van der Molen L van Son RJJH de Bree R Al-Mamgani A. Dysphagia, Trismus and Speech Impairment Following Radiation-Based Treatment for Advanced Stage Oropharyngeal Carcinoma: A One-Year Prospective Evaluation. Eur Arch Otorhinolaryngol (2022) 279(2):1003–27. doi: 10.1007/s00405-021-06870-x 34043065

[82] Surov A Wienke A. Low Skeletal Muscle Mass Predicts Relevant Clinical Outcomes in Head and Neck Squamous Cell Carcinoma A Meta Analysis. Ther Adv Med Oncol (2021) 13:17588359211008844. doi: 10.1177/17588359211008844 PMC8127787 34035838

[83] Bril SI Pezier TF Tijink BM Janssen LM Braunius WW de Bree R. Preoperative Low Skeletal Muscle Mass as a Risk Factor for Pharyngocutaneous Fistula and Decreased Overall Survival in Patients Undergoing Total Laryngectomy. Head Neck (2019) 41(6):1745–55. doi: 10.1002/hed.25638 PMC6590286 30663159

[84] Jung AR Roh JL Kim JS Choi SH Nam SY Kim SY. The Impact of Skeletal Muscle Depletion on Older Adult Patients With Head and Neck Cancer Undergoing Primary Surgery. J Geriatr Oncol (2021) 12(1):128–33. doi: 10.1016/j.jgo.2020.06.009 32565144

[85] Alwani MM Jones AJ Novinger LJ Pittelkow E Bonetto A Sim MW. Impact of Sarcopenia on Outcomes of Autologous Head and Neck Free Tissue Reconstruction. J Reconstr Microsurg (2020) 36(5):369–78. doi: 10.1055/s-0040-1701696 32088918

[86] Jones AJ Campiti VJ Alwani M Novinger LJ Bonetto A Sim MW. Skeletal Muscle Index's Impact on Discharge Disposition After Head and Neck Cancer Free Flap Reconstruction. Otolaryngol Head Neck Surg (2021) 165(1):59–68. doi: 10.1177/0194599820973232 33290190

[87] Jones AJ Campiti VJ Alwani M Novinger LJ Tucker BJ Bonetto A. Sarcopenia is Associated With Blood Transfusions in Head and Neck Cancer Free Flap Surgery. Laryngoscope Investig Otolaryngol (2021) 6(2):200–10. doi: 10.1002/lio2.530 PMC8035950 33869752

[88] Ansari E Chargi N van Gemert JTM van Es RJJ Dieleman FJ Rosenberg AJWP. Low Skeletal Muscle Mass is a Strong Predictive Factor for Surgical Complications and a Prognostic Factor in Oral Cancer Patients Undergoing Mandibular Reconstruction With a Free Fibula Flap. Oral Oncol (2020) 101:104530. doi: 10.1016/j.oraloncology.2019.104530 31881447

[89] Wong A Zhu D Kraus D Tham T. Radiologically Defined Sarcopenia Affects Survival in Head and Neck Cancer: A Meta-Analysis. Laryngoscope (2021) 131(2):333–41. doi: 10.1002/lary.28616 32220072

[90] Takenaka Y Takemoto N Oya R Inohara H. Prognostic Impact of Sarcopenia in Patients With Head and Neck Cancer Treated With Surgery or Radiation: A Meta-Analysis. PloS One (2021) 16(10):e0259288. doi: 10.1371/journal.pone.0259288 34714876 PMC8555817

[91] Findlay M White K Stapleton N Bauer J. Is Sarcopenia a Predictor of Prognosis for Patients Undergoing Radiotherapy for Head and Neck Cancer? A Meta-Analysis. Clin Nutr (2021) 40(4):1711–8. doi: 10.1016/j.clnu.2020.09.017 32994071

[92] Chou WC Chang PH Chen PT Wang HM Yeh KY Lu CH. Clinical Significance of Vulnerability Assessment in Patients With Primary Head and Neck Cancer Undergoing Definitive Concurrent Chemoradiation Therapy. Int J Radiat Oncol Biol Phys (2020) 108(3):602–11. doi: 10.1016/j.ijrobp.2020.01.004 31987971

[93] Bras L de Vries J Festen S Steenbakkers RJHM Langendijk JA Witjes MJH. Frailty and Restrictions in Geriatric Domains are Associated With Surgical Complications But Not With Radiation-Induced Acute Toxicity in Head and Neck Cancer Patients: A Prospective Study. Oral Oncol (2021) 118:105329. doi: 10.1016/j.oraloncology.2021.105329 34111770

[94] Fu TS Sklar M Cohen M de Almeida JR Sawka AM Alibhai SMH. Is Frailty Associated With Worse Outcomes After Head and Neck Surgery? A Narrative Review. Laryngoscope (2020) 130(6):1436–42. doi: 10.1002/lary.28307 31633817

[95] Mendes ML Mahl C Carvalho AC Santos VS Tanajura DM Martins-Filho PR. Frailty and Risk of Complications in Head and Neck Oncologic Surgery. Systematic Review and Dose-Response Meta-Analysis. Med Oral Patol Oral Cir Bucal (2021) 26(5):e582–9. doi: 10.4317/medoral.24588 PMC8412444 34414998

[96] Voora RS Qian AS Kotha NV Qiao EM Meineke M Murphy JD. Frailty Index as a Predictor of Readmission in Patients With Head and Neck Cancer. Otolaryngol Head Neck Surg (2021) 1945998211043489. doi: 10.1177/01945998211043489 34520305

[97] de Vries J Bras L Sidorenkov G Festen S Steenbakkers RJHM Langendijk JA. Frailty is Associated With Decline in Health-Related Quality of Life of Patients Treated for Head and Neck Cancer. Oral Oncol (2020) 111:105020. doi: 10.1016/j.oraloncology.2020.105020 33045628

[98] Hamaker ME Jonker JM de Rooij SE Vos AG Smorenburg CH van Munster BC. Frailty Screening Methods for Predicting Outcome of a Comprehensive Geriatric Assessment in Elderly Patients With Cancer: A Systematic Review. Lancet Oncol (2012) 13:e437–44. doi: 10.1016/S1470-2045(12)70259-0 23026829

[99] Noor A Gibb C Boase S Hodge JC Krishnan S Foreman A. Frailty in Geriatric Head and Neck Cancer: A Contemporary Review. Laryngoscope (2018) 128(12):E416–24. doi: 10.1002/lary.27339 30329155

[100] Pottel L Lycke M Boterberg T Pottel H Goethals L Duprez F. Serial Comprehensive Geriatric Assessment in Elderly Head and Neck Cancer Patients Undergoing Curative Radiotherapy Identifies Evolution of Multidimensional Health Problems and is Indicative of Quality of Life. Eur J Cancer Care (Engl) (2014) 23(3):401–12. doi: 10.1111/ecc.12179 24467393

[101] Bruijnen CP Heijmer A van Harten-Krouwel DG van den Bos F de Bree R Witteveen PO. Validation of the G8 Screening Tool in Older Patients With Cancer Considered for Surgical Treatment. J Geriatr Oncol (2021) 12(5):793–8. doi: 10.1016/j.jgo.2020.10.017 33172806

[102] Williams GR Deal AM Muss HB Weinberg MS Sanoff HK Guerard EJ. Frailty and Skeletal Muscle in Older Adults With Cancer. J Geriatr Oncol (2018) 9(1):68–73. doi: 10.1016/j.jgo.2017.08.002 28844849 PMC5742058

[103] Dunne RF Roussel B Culakova E Pandya C Fleming FJ Hensley B. Characterizing Cancer Cachexia in the Geriatric Oncology Population. J Geriatr Oncol (2019) 10(3):415–9. doi: 10.1016/j.jgo.2018.08.008 PMC6401352 30196027

[104] Mascarella MA Gardiner L Patel T Vendra V Khan N Kergoat MJ. Cervical Paraspinal Skeletal Muscle Index Outperforms Frailty Indices to Predict Postoperative Adverse Events in Operable Head and Neck Cancer With Microvascular Reconstruction. Microsurgery (2022) 42(3):209–16. doi: 10.1002/micr.30848 34935198

[105] Meerkerk CDA Chargi N de Jong PA van den Bos F de Bree R. Low Skeletal Muscle Mass Predicts Frailty in Elderly Head and Neck Cancer Patients. Eur Arch Otorhinolaryngol (2022) 279(2):967–77. doi: 10.1007/s00405-021-06835-0 PMC8794912 33956205

